# High prevalence of uncontrolled hypertension among patients with type 2 diabetes mellitus: a hospital-based cross-sectional study in Southwestern Uganda

**DOI:** 10.11604/pamj.2021.39.142.28620

**Published:** 2021-06-21

**Authors:** Patrick Kambale Saasita, Siraj Senoga, Kakule Muhongya, David Collins Agaba, Richard Migisha

**Affiliations:** 1 Department of Internal Medicine, Mbarara University of Science and Technology, Mbarara, Uganda,; 2 Department of Accident and Emergency, Kampala International University Western campus, Ishaka, Bushenyi, Uganda,; 3 Department of Physiology, Mbarara University of Science and Technology, Mbarara, Uganda

**Keywords:** Uncontrolled hypertension, diabetes, prevalence, Uganda

## Abstract

**Introduction:**

most patients with diabetes mellitus are prone to uncontrolled blood pressures despite effective medical therapies; only about 30% of hypertensive patients have their blood pressures controlled. Poor control of hypertension is associated with increased risk for cardiovascular mortality and morbidity. We aimed to determine the prevalence and associated factors of uncontrolled hypertension among patients with type 2 diabetes mellitus (T2DM) attending ambulatory care at Mbarara Regional Referral Hospital, Southwestern Uganda.

**Methods:**

we conducted a cross-sectional study from January to April 2019, among hypertensive T2DM patients. We used a structured questionnaire to obtain data on socio-demographic and clinical characteristics. We defined uncontrolled hypertension in participants with blood pressure ≥140/90mmHg and performed binary logistic regression to determine factors associated with uncontrolled hypertension.

**Results:**

we analyzed data of 206 hypertensive participants with concomitant T2DM, with a median age of 54 (IQR, 49-60) years, and median duration of diabetes of 4 (IQR, 3-8) years; 71% were female. The prevalence of uncontrolled hypertension was 82.5% (170/206). Isolated systolic hypertension (aOR=7.58; 95%CI: 2.18-26.36, P=0.001) and left ventricular hypertrophy (LVH) (aOR=5.38; 95%CI: 1.11-26.10, P=0.037) were significantly associated with uncontrolled hypertension.

**Conclusion:**

this study revealed a high prevalence of uncontrolled hypertension among T2DM patients in Southwestern Uganda. Isolated systolic hypertension and LVH were the key factors associated with uncontrolled hypertension. We recommend optimization therapy to reduce the burden of uncontrolled hypertension among patients with T2DM especially in those with isolated systolic hypertension, and left ventricular hypertrophy, who are at higher cardiovascular risk.

## Introduction

The burden of diabetes has reached epidemic proportions globally, with an estimated 500 million individuals lining with the disease [1]. The prevalence of diabetes is expected to increase more in low-income countries than high-income countries [2,3]. Cross-sectional surveys done in Uganda have reported prevalence estimates of Diabetes Mellitus (DM) ranging from 2% to 8% [4,5]. Hypertension is the major cause of cardiovascular disease (CVD) and deaths worldwide, accounting for an estimated 7.5 million deaths annually [6]. The co-existence of hypertension and DM ranges from 20 to 60%, and varies with ethnicity, body mass index and age [7]. Nonetheless, adequate treatment of hypertension may reduce cardiovascular-related mortality and morbidity [8]. Hypertension is the strong predictor of adverse cardiovascular outcomes (coronary artery disease, heart failure and stroke) in individuals with diabetes [9], with up to 75% of cardiovascular diseases attributed to hypertension [10] in this patient population. In addition, sub-optimal control of hypertension accelerates the development and progression of both micro-and macro-vascular complications among individuals with diabetes. Quantifying the burden of uncontrolled hypertension, and identifying its associated factors will provide useful information for evidence-based recommendations in addressing control of hypertension in diabetic individuals. There are limited data on the burden of uncontrolled hypertension and its associated factors in Uganda. We aimed to determine the prevalence and associated factors of uncontrolled hypertension among patients with T2DM at Mbarara regional referral hospital (MRRH) in Southwestern Uganda. Our study adopted the Joint national committee (JNC8) guidelines [11] to describe uncontrolled hypertension in our study population. We hypothesized that sociodemographic and clinical factors were associated with uncontrolled hypertension, and tested this hypothesis in a cross-sectional survey.

## Methods

**Study setting, study design, and study population:** we conducted a cross-sectional study within a cohort of patients with diabetes followed up at MRRH diabetes and endocrinology clinic in Southwestern Uganda. Our study participants were patients who consented to participate in the Cardiovascular autonomic neuropathy (CAN) study. The methods for this study have been described in detail elsewhere [12]. Briefly, the CAN study recruited 303 participants aged 18-65 years, through consecutive sampling. The study excluded patients with multi-morbidities such as: electrolyte imbalances, renal, hepatic and endocrine disorders. The present study included CAN study participants with T2DM aged 35 years and above with documented history of hypertension and those fulfilling the JNC8 criteria for hypertension (systolic (BP≥140mmHg and/or diastolic BP≥90mmHg) considering the mean of at least the two last blood pressure(BP) measurements taken during the two last visits. Our analysis included only participants with concomitant hypertension and T2DM (N=206). We excluded participants who were on follow-up for less than one month from the time of diagnosis of diabetes.

**Data collection and study definitions:** we administered a structured questionnaire and reviewed patients´ medical records. The data were collected from January to April 2019. Data on sociodemographic, clinical and laboratory characteristics were collected. The sociodemographic data included: age, sex, tobacco use, and alcohol use. Clinical parameters included: blood pressure, body weight, height, duration of diabetes, glycemic control, mode of antihypertensive therapy, and electrocardiogram (ECG) changes. We defined history of smoking in participants who smoked at least one cigarette per day for at least one year. Twelve lead ECG recordings were performed for all our study participants using a portable ECG machine (Edan Instruments, Inc., Hessen, Germany). The different ECG abnormalities were defined using Minnesota criteria. Left ventricle hypertrophy was defined by Sokolow-Lyon criteria whereby amplitude of the S wave in V1 plus amplitude of the R wave in V5 or V6 (using the tallest R wave) is greater than 35mm. Weight and height were measured to the nearest 0.1kg and 0.1cm, respectively, with participants putting on light clothes and no shoes. Body mass index (BMI) was calculated as body weight in kilograms divided by the square of the body height in meters. We used a recognized criteria to categorize BMI as normal, underweight, overweight and obese [13]. We collected venous blood samples from the antecubital vein for glycosylated haemoglobin (HbA1c) using a becton, dickinson(BD) Vacutainer tube containing EDTA (becton, Dickinson and Co, Franklin Lakes, NJ, USA). HbA1c was measured at Lancet laboratories using an automated high performance liquid chromatography analyser (Cobas integra 400, Roche diagnostics, Basel, Switzerland), according to the standard operating procedure of the International Federation of Clinical Chemistry and Laboratory Medicine (IFCC) [14]. Blood pressure was recorded in a sitting position using an automatic sphygmomanometer in the upper arm (Omron HEM 705 LP, Omron Healthcare, Inc., Bannockburn, IL, USA), with arm at the level of the heart and the feet together, after the participant had rested for at least five minutes. Diagnosis of hypertension and types of antihypertensive medications were captured from medical records. We defined uncontrolled hypertension in participants with systolic blood pressure greater than or equal to 140mmHg and/or diastolic blood pressure of greater than or equal to 90mmHg, in accordance with the BP treatment targets recommended by the Eight Joint National Committee Criteria (2014) [11].

**Sample size and statistical analyses:** the parent study required a sample size of 296 participants to enable a 5% precision with a 95% confidence interval around a prevalence estimate of CAN of 20% [15]. Data were entered into Excel then exported to Stata version 14 (StataCorp, College Station, Texas, USA) for all analyses. Prevalence of uncontrolled hypertension was determined as the proportion of study participants meeting the criteria for uncontrolled hypertension. Socio-demographic and clinical characteristics were described as frequencies and percentages for categorical variables (sex, tobacco use, alcohol use, ECG changes). Continuous variables were described as means (±standard deviation) for normally distributed variables (HbA1c for glycated hemoglobin) and median (+ Inter-quartile range) for non-normally distributed variables (age and duration of diabetes). We then performed univariable and multivariable logistic regression to identify socio-demographic and clinical factors associated with uncontrolled hypertension. Variables with a P-value ≤ 0.4 in univariable models were included in multivariable models through backward stepwise method. Variables in the final model with p<0.05, were considered statistically significant.

**Ethical considerations:** our study was approved by the institutional ethics review board of Mbarara University of Science and Technology (MUST-REC). The study also received clearance for the study from the Uganda National Council of science and technology (UNCST) and from the Research Secretariat in the Office of the President of Uganda, in accordance with the national guidelines. All study participants provided written informed consent before recruitment and participation. Participants who could not write indicated consent with a thumb print.

## Results

**Figure 1:**
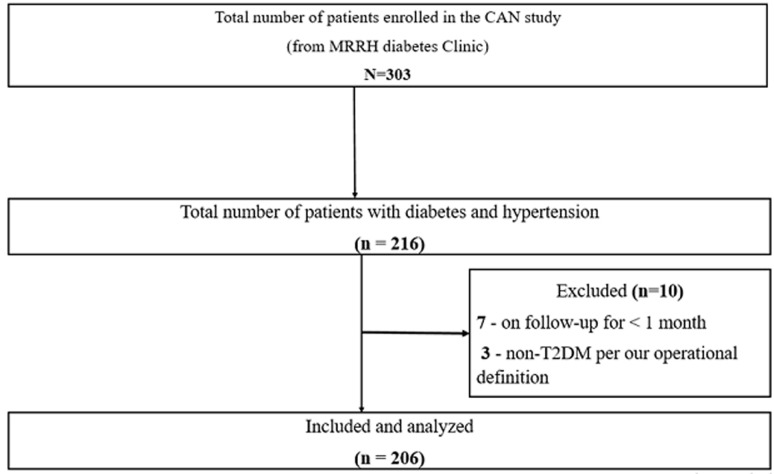
flow chart for recruitment of study participants, January-April 2019

Of the 303 participants enrolled in the CAN study, we present results for 206 participants included into the current study. The reasons for exclusion are presented in Figure 1.

**Baseline demographic and clinical characteristics:** the baseline demographic and clinical characteristics of study participants are presented in Table 1. Most of the study participants were female (71.36%). The median age was 54 years (IQR, 49-60 years) with a median duration of diabetes of 4 years (IQR, 2-8 years). The mean Spontaneous bacterial peritonitis (SBP) and diastolic blood pressure (DBP) were 149.61±19.98mmHg and 89.65±10.44mmHg respectively. The mean HbA1c of 9.52±2.42%. The proportion of isolated systolic hypertension was 34.47%. Almost half of our patients were on a single antihypertensive drug (49.51%). Electrocardiogram changes were found in 110/206 (55.40%) of participants, with ST segment and Q wave abnormalities being the most common ECG change found in 70/206 (33.98%) of the participants.

**Table 1:** baseline demographic and clinical characteristics of study participants

Baseline characteristics	Frequency (N=206)
Female sex, n (%)	147 (71.36)
Median age (IQR) in years	54 (49 - 60)
Median duration of DM (IQR) in years	4 (2 - 8)
**Alcohol intake, n (%)**	
Yes	19 (9.22)
No	187 (90.78)
**Smoking, n (%)**	
Yes	10 (5.85)
No	168 (95.15)
**BMI (Kg/m^2^), n (%)**	
Normal BMI (18.5-24.9)	72 (34.95)
Overweight (25-29.9)	80 (38.83)
Obese (≥ 30), n (%)	54 (26.21)
**Mean BP (mmHg) (± SD)**	
SBP	149.61 (±19.98)
DBP	89.65 (±10.44)
Isolated systolic hypertension, n (%)	71 (34.47)
Mean HbA1c (±SD)	9.52 (±2.42)
**Mode of treatment, n (%)**	
Monotherapy	102 (49.51)
More than one drug	72 (34.95)
Not on any antihypertensive	32 (15.53)
**Presence of ECG changes, n (%)**	
Any ECG changes	110 (53.40)
LVH	35 (16.99)
ST abnormalities and Q waves	70 (33.98)
Others	43 (20.87)

SD: standard deviation; SBP: systolic blood pressure; DBP: diastolic blood pressure; HbA1c: glycosylated hemoglobin; BMI: body mass index; IQR: inter-quartile range; DM: diabetes mellitus; ECG: electrocardiograph; LVH: left ventricular hypertrophy

**Prevalence of uncontrolled hypertension:** among the 206 participants assessed for BP control, 170 met the definition of uncontrolled hypertension for a prevalence of82.5% (95%CI: 76.7- 87.2%); only 36 (17.5%) participants had controlled BP.

**Factors associated with uncontrolled hypertension:** demographic and clinical factors associated with uncontrolled hypertension are shown in Table 2. In the multivariable model, isolated systolic hypertension (aOR=7.58; 95%CI: 2.18-26.36, P= 0.001) and Left Ventricular Hypertrophy (aOR=5.38; 95%CI: 1.11-26.10, P= 0.037) were significantly associated with uncontrolled hypertension among the study participants. There was no association with HbA1c, BMI, mode of antihypertensive therapy and ECG changes except left ventricular hypertrophy (LVH).

**Table 2:** factors associated with uncontrolled hypertension in among study participants

	% Uncontrolled hypertension	Unadjusted analysis		Adjusted analysis	
**Characteristic**	**n/N (%)**	**OR(95% CI**)	**P-value**	**Adjusted OR (95%CI)**	**P value**
**Age in years**					
18-44	14/21 (66.67)	Ref		Ref	
45-65	156/185 (84.32)	2.69 (0.99 - 7.23)	0.050	2.21 (0.72 - 6.80)	0.165
**Sex**					
Male	48/59 (81.36)	Ref			
Female	122/147 (82.99)	1.12 (0.51 - 2.55)	0.780	-	-
**Duration of DM**					
≤10 years	132/160 (82.50)	Ref			
>10 years	38/46 (82.61)	1.00 (0.42 - 2.39)	0.986	-	-
**BMI (in Kg/m^2^)**					
Normal (18.5-24.9)	58/72 (80.56)	Ref		Ref	
Overweight (25-29.9)	64/80 (80.00)	0.96 (0.43 - 2.14)	0.932	0.99 (0.41 - 2.39)	0.989
Obese (≥30)	48/54 (88.89)	1.93 (0.69 - 5.40)	0.210	2.37 (0.75 - 7.03)	0.145
**Alcohol intake**					
No	155/187 (82.89)	Ref			
Yes	15/19 (78.95)	0.77 (0.48 - 2.64)	0.667	-	-
**Smoking**					
No	162/196 (82.65)	Ref			
Yes	8/10 (80.00)	0.84 (0.17 - 4.13)	0.830	-	-
**HbA1c**					
<7%	27/30 (90.00)	Ref		Ref	
≥7%	143/176 (81.25)	0.48 (0.14 - 1.68)	0.252	0.34 (0.09 - 1.31)	0.117
**Isolated systolic hypertension**					
No	102/135 (75.56)	Ref		Ref	
Yes	68/71 (95.77)	7.33 (2.16 - 24.86)	0.001	7.19 (2.06 - 25.04)	**0.002**
**Mode of antihypertensive treatment**					
At least two drugs	59/73 (80.82)	Ref			
Monotherapy or none	111/133 (83.46)	0.19 (0.57 - 2.51)	0.634	-	-
**Presence of any ECG changes**					
No	76/96 (79.17)	Ref			
Yes	94/110 (85.45)	1.55 (0.75 - 3.18)	0.238	-	-
**Left Ventricular Hypertrophy (LVH)**					
No	137/171 (80.12)	Ref		Ref	
Yes	33/35 (94.29)	4.09 (0.94 - 17.91)	0.061	5.67 (1.16 - 27.70)	**0.032**
**ST changes and Q waves**					
No	114/136 (83.82)	Ref		Ref	
Yes	56/70 (80.00)	1.55 (0.75 - 3.19)	0.238	0.53 (0.22 - 1.25)	0.148


Ref: Reference category; CI: Confidence interval; OR: Odds ratio; BMI: Body mass index

## Discussion

This study established a high burden of uncontrolled hypertension, detected in eight of every ten hypertensive patients with concomitant T2DM, in Southwestern Uganda. This is higher in comparison to studies done in cohorts of hypertensive individuals elsewhere [16-18]. The high prevalence of uncontrolled hypertension of 82.52% in this hospital-based study is similar to findings from other studies done in Africa. For instance, similar alarmingly high rates of: 82.8%, 91.1%, 75.5%, and 84.5%, have been reported in Morocco [19], Ghana [20], South Africa [21], and Tanzania [22], respectively among hypertensive individuals. These data add more weight to the notion that, the number of individuals with uncontrolled hypertension has risen over the recent past, with the highest being in Africa (70% uncontrolled vs. 30% controlled), as indicated by data from World Health Organization [23]. It is worth mentioning, that, half of the participants in the current study were treated with a single antihypertensive agent (49.5%) and some (15%) were not on any antihypertensive medications. This could partly explain the high prevalence of uncontrolled hypertension in our study population. A combination of two or more drugs seems to be inevitable, because resistant hypertension is a common finding in diabetes [24]. Thiazide and thiazide-like diuretics might be beneficial, alone or in a fixed-dose combination with angiotensin converting enzyme inhibitors (ACEIs) or angiotensin receptor blockers (ARBs) [25]. Another plausible explanation for the high prevalence of uncontrolled hypertension in this study, could be failure to optimize antihypertensive medications in our study population, given the fact that combination therapy did not achieve the blood pressure control. Isolated systolic hypertension and LVH were the significant factors associated with uncontrolled hypertension. Previous studies have demonstrated the great challenge of blood pressure control and high risk of cardiovascular complications related to poor response to treatment among patients with isolated systolic hypertension [26]. The prevalence of isolated systolic hypertension is high among patients with T2DM, especially those with old age [27]. Atherosclerosis and diabetes are among the most common causes of isolated systolic hypertension [28].

We established an association between LVH and uncontrolled hypertension in this study. This may be because of reverse causation, where LVH is a consequence of the poorly controlled blood pressure and T2DM. The LVH in patients with uncontrolled hypertension, arises as a result of a compensatory mechanism due to increased pressure overload to the heart [29]. Furthermore, the presence of T2DM has been found to be associated with increased risk of LVH in a multiethnic population-based study [30]. Moreover, patients with LVH have an elevated risk for cardiovascular disease (CVD) and mortality, regardless of the presence of hypertension and diabetes [31,32]. Thus, in diabetic individuals this risk may be amplified due to accelerated development of LVH [33]. These data give cause to regularly screen for CVD among diabetics, even more so, in those with concomitant hypertension, so as to avert premature mortality and morbidity from CVD in this population. Overall, our study findings have clinical and public health implications with regard to care for individuals with diabetes and coexisting hypertension. First, the high occurrence of uncontrolled hypertension in this study population supports an urgent need for quality improvement efforts aimed at achieving optimal control of blood pressure in this patient population, including optimization of therapy for antihypertensive medications; moreover, sub-optimal control of hypertension accelerates the development and progression of both micro-and macro-vascular complications among individuals with diabetes [34].

However, further studies to assess the clinical and prognostic implications of uncontrolled hypertension in this study population are warranted. Second, our study findings support an urgent need for routine and systematic screening of high blood pressure in diabetic individuals, given the high prevalence of uncontrolled hypertension; this will help in timely detection and appropriate management of uncontrolled hypertension, and consequently minimize the risk of morbidity and mortality from CVD in this patient population. Additionally, some authors have previously observed that clinicians tend to concentrate at achieving glycemic control at the expense of blood pressure control [35]; others have highlighted other possible causes of uncontrolled hypertension including clinicians´ inertia, and poor patient adherence [36]. We therefore recommend a follow-up qualitative study to further elucidate the other factors associated with uncontrolled hypertension in our Ugandan setting; this will better inform quality improvement efforts, and interventions targeting improved care of a diabetic hypertensive. Our study has some limitations: First, this study was conducted in a regional referral hospital for Southwestern region of Uganda, and therefore referral bias may overestimate the prevalence of uncontrolled hypertension observed in our study. Second, some of the measures used in the study, such as: alcohol and tobacco use, were self-reported, and may be prone to social desirability bias. Nonetheless, we cross-checked these measures with information already provided in the medical records, including their data on medication use, and clinical history. Third, we did not assess some of the factors that are known to affect BP control such as: patients' adherence and health-system factors. Thus, our findings are prone to residual confounding from these factors that we did not adjust for in our analysis. Finally, we cannot assign the time directionality of association between the independent factors (e.g. LVH, isolated systolic hypertension) and uncontrolled hypertension itself, because of the cross-sectional nature of our study design. This can be explored in longitudinal studies. Despite these limitations, our study is among the initial studies to provide useful epidemiological data quantifying burden of uncontrolled hypertension among individuals with diabetes in ambulatory care in Uganda, and identified some of the clinical factors associated with poor blood pressure control in this patient population.

## Conclusion

Our study revealed a high prevalence of uncontrolled hypertension, detected in more than three-quarters of patients with T2DM in ambulatory diabetes care in Uganda. Isolated systolic hypertension and LVH were key factors associated with uncontrolled hypertension There is an urgent need for early recognition and appropriate management of high blood pressure in patients with concomitant T2DM. We recommend optimization of the therapy for blood pressure control in this patient population, especially in those with isolated systolic hypertension and those with LVH, who are at higher cardiovascular risk.

**Funding:** this study was supported by Förderverein UNIKIN also known as BEBUC (Bourse d´Excellence Bringmann aux Universités Congolaises), and the Fogarty International center and co-founding partners (NIH Common Fund, office of strategic coordination, office of the director (OD/OSC/CF/NIH); office of AIDS research, office of the director (OAR/NIH); National Institute of mental health (NIMH/NIH); and National Institute of Neurological disorders and stroke (NINDS/NIH)) of the National Institutes of Health under award number D43TW010128. The content is solely the responsibility of the authors and does not necessarily represent the official views of the National institutes of health.

### What is known about this topic

- High prevalence of uncontrolled hypertension across the world in the general population and among diabetes patients;
- Risk factors of uncontrolled hypertension (old age, poor compliance to therapy, comorbidities like diabetes, kidney disease);
- Rise of uncontrolled hypertension prevalence in Africa.

### What this study adds

- It highlights a high prevalence of uncontrolled hypertension in Uganda where there is a lack of data about control of hypertension especially among T2DM patients;
- It shows cardiac risk related to uncontrolled hypertension evidenced by the presence of LVH detected in almost 1 of every 5T2DM patients with hypertension;
- It emphasizes on the role of optimization therapy for the patients at higher risk.
